# Inhibiting DNA methylation switches adipogenesis to osteoblastogenesis by activating Wnt10a

**DOI:** 10.1038/srep25283

**Published:** 2016-05-03

**Authors:** Yii-Shyuan Chen, Rui Wu, Xiaosong Yang, Shuping Kou, Ormond A. MacDougald, Liqing Yu, Hang Shi, Bingzhong Xue

**Affiliations:** 1 Center for Obesity Reversal, Department of Biology, Georgia State University, Atlanta, GA, USA; 2 Department of Molecular & Integrative Physiology, University of Michigan Medical School, Ann Arbor, MI, USA; 3 Department of Animal and Avian Sciences, University of Maryland, College Park, Maryland, USA

## Abstract

Both adipocytes and osteoblasts share the mesodermal lineage that derives from mesenchymal stem cells. Most studies investigating the mechanisms underlying the regulation of adipogenic or osteoblastogenic development focus on transcriptional pathways; little is known about the epigenetic mechanisms in this process. We thus determined the role of 5-aza-2′-deoxycytidine (5-Aza-dC), an inhibitor of DNA methylation, in the lineage determination between adipogenesis and osteoblastogenesis. Inhibiting DNA methylation in 3T3-L1 preadipocytes by 5-Aza-dC significantly inhibited adipogenesis whereas promoted osteoblastogenesis. This dual effect of 5-Aza-dC was associated with up-regulation of Wnt10a, a key factor determining the fate of the mesenchymal lineage towards osteoblasts. Consistently, IWP-2, an inhibitor of Wnt proteins, was found to prevent the anti-adipogenic effect of 5-Aza-dC in 3T3-L1 preadipocytes and block the osteoblastogenic effect of 5-Aza-dC in ST2 mesenchymal stem cell line. Finally, the Wnt10a 5′-region is enriched with CpG sites, whose methylation levels were markedly reduced by 5-Aza-dC. Thus we conclude that inhibiting DNA methylation by 5-Aza-dC mutual-exclusively regulates the lineage determination of adipogenesis and osteoblastogenesis by demethylating Wnt10a gene and upregulating its expression. Our study defines DNA methylation as a novel mechanism underlying adipocyte and bone cell development.

Both adipocytes and osteoblasts share the mesodermal lineage that derives from mesenchymal stem cells^1^,^2^. Extensive studies have been devoted to the investigation of the pathways mediating the formation of adipocytes and osteoblasts over years. Adipogenesis is highly regulated by a sequential cascade of transcriptional events^3^. Key transcriptional factors in this transcriptional program controlling adipogenesis include several members of CCAAT/enhancer-binding protein (C/EBP) family including C/EBPα, β and δ, and the nuclear receptor peroxisome proliferator γ (PPARγ)^3^. The initiation of adipogenesis is caused by early induction of C/EBPβ and δ. These early transcriptional factors subsequently activate two key transcriptional factors PPARγ and C/EBPα, interaction of which leads to the late determination of the adipocyte phenotype by inducing a variety of adipocyte phenotypic genes such as adipocyte protein 2 (aP2), glucose transporter 4 (GLUT4), etc^3^. On the other hand, a number of transcriptional repressors have also been identified to counter-regulate the pro-adipogenic factors in the process of adipogenesis, including GATA2/3, chicken ovalbumin upstream promoter transcription factor (COUP-TF), interferon regulatory factors (IRFs), and Wnt family proteins^3^,^4^,^5^,^6^,^7^. The differentiation process that determines the adipocyte fate is highly regulated by a coordinated control of these positive and negative transcriptional factors.

Wnt signaling is a key determinant of the fate between adipogenic and osteoblastogenic cells. Wnt signaling includes 1) the canonical Wnt pathway, 2) the noncanonical Wnt and calcium regulation pathway, and 3) the noncanonical cell polarity regulation pathway^8^. In the canonical Wnt pathway, also known as the Wnt/β-catenin pathway, Wnt family proteins bind to the Frizzled family receptors to regulate the cytosolic stability of β-catenin, a coactivator of TCF/LEF family transcriptional factors involved in various biological functions such as cell proliferation and development^8^. Without the presence of Wnt proteins (i.e., Wnt10a), β-catenin, a component of a repressing complex comprised of Axin, adenomatosis polyposis coli (APC), glycogen synthase kinase 3 (GSK3) and casein kinase 1α (CK1α), is subjected to phosphorylation by the kinases GSK3 and CK1α within the complex, and is subsequently programmed for ubiquitin-associated proteosomal degradation^9^. With the presence of the Wnt proteins, β-catenin can be stabilized in the cytosol by dissociation from the repressing complex, translocate into the nucleus, and co-activate the transcriptional factors TCF/LEF, which in turn promotes transcription of target genes^9^. Wnt signaling pathways are highly conserved and have been extensively investigated organismal development, cancer, and stem cell biology^8^. Research has established the Wnt/β-catenin signaling as a key determinant of the fate between adipogenic and osteobalstogenic lineages^5^,^10^. For example, Activation of Wnt signaling suppresses adipogenesis by inhibiting PPARγ and C/EBPα^5^. Wnt family proteins have also been shown to exert a coordinated control over inhibition of adipogenesis and stimulation of osteoblastogenesis via a Wnt/β-catenin-dependent mechanism^10^.

Most studies investigating the mechanisms underlying the regulation of adipogenesis and osteoblastogenesis focus on transcriptional pathways; little is known about the epigenetic mechanisms in this process. Epigenetic regulation, including DNA methylation, is a molecular link between environmental factors (e.g. diets) and complex diseases, including obesity and diabetes. DNA methylation of cytosines at primarily CpG dinucleotides is the most common epigenetic modification. CpGs are often enriched in the promoter and the first exon/5′-untranslated region of genes^11^. *De novo* methylation is mediated by DNA methyltransferase (DNMT) 3a and 3b. Once established, DNA methylation is then maintained through mitosis primarily by the maintenance enzyme DNMT1^12^. Promoters of transcriptionally active genes are typically hypo-methylated^13^, whereas DNA hyper-methylation can result in gene silencing by affecting the binding of methylation-sensitive DNA binding proteins and/or by interacting with various histone modifications and co-repressors that alter DNA accessibility to transcriptional factors^13^. Evidence converges to suggest that epigenetic events figure prominently in adipogenesis^14^,^15^. This is a new emerging research area; however, much remains to be discovered on how epigenetic mechanisms regulate lineage determination of adipogenesis vs. osteoblastogenesis.

5-Aza-dC is a nucleoside-based DNA methyltransferase inhibitor widely used to study the role of DNA methylation in regulation of cell development and cancer^16^,^17^. In the present study, we aimed to determine the role of 5-aza-2′-deoxycytidine (5-Aza-dC), an inhibitor of DNA methylation, in regulation of adipogenesis and osteoblastogenesis. We characterized the adipogenic and osteoblastogenic phenotypes in 3T3-L1 preadipocytes and ST2 mesenchymal stem cells where the DNA methylation was pharmacologically inhibited by 5-Aza-dC. We then explored the molecular mechanism underlying the phenotypic changes of these cells by inhibition of DNA methylation.

## Results

### Inhibition of DNA methylation by 5-Aza-dC at the early stage of differentiation suppresses 3T3-L1 adipogenesis

**Figure 1:**
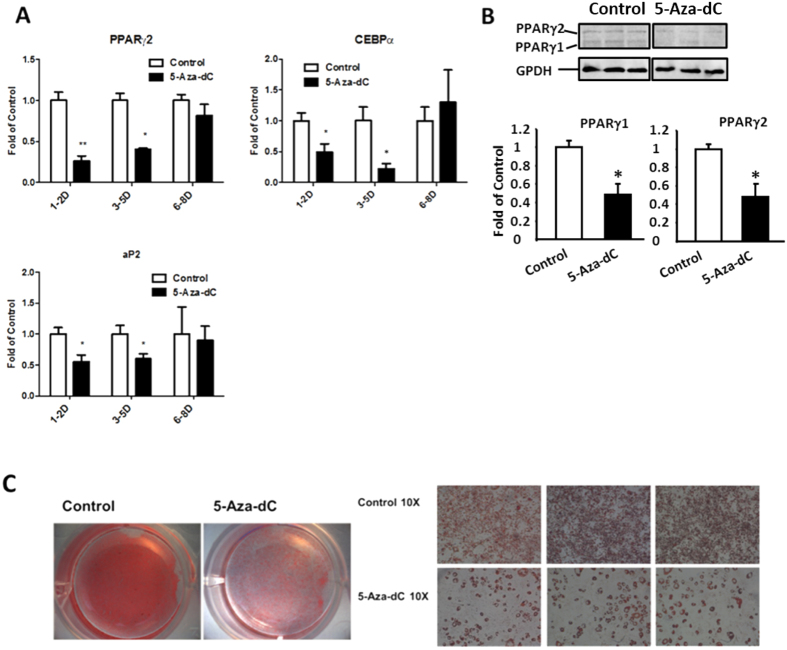
Inhibition of DNA methylation by 5-Aza-dC at the early stage of differentiation suppresses 3T3-L1 adipogenesis. (**A**) Treatment of 5-Aza-dC at the early differentiation stage from day 1–5 inhibits decreases mRNA expression of adipogenic markers such as PPARγ2, CEBPα, and aP2. (**B**) Treatment of 5-Aza-dC at the early differentiation stage of day 1–2 decreases PPARγ2 protein expression. (**C**) Oil Red O staining reveals less lipid accumulations in 3T3-L1 cells treated with 5-Aza-dC at the early differentiation stage of day 1–2. 3T3-L1 preadipocyte were induced to differentiate with a differentiation medium as described in the Materials and Methods, and were concurrently treated with either PBS or 5-Aza-dC at day 1–2, 3–5 and 6–8 as indicated in the figure and cells were harvested at day 8 of the differentiation for the measurement of gene expression. mRNA and protein levels were measured by real-time RT-PCR and immunoblotting, respectively. Oil Red O staining were performed as described in Materials and Methods. Representative blots and images are shown in (**B**,**C**), respectively. All data are expressed as mean ± SEM (n = 3–6); *p < 0.05; **p < 0.01 vs. control.

We employed a pharmacological approach to inhibit DNA methylation during 3T3-L1 differentiation. 5-Aza-dC is a nucleoside-based DNA methyltransferase inhibitor widely used to study the role of DNA methylation in the regulation of cell development and cancer^16^,^17^. 3T3-L1 preadipocytes were induced to differentiate with a differentiation medium and were concurrently treated with either PBS or 5-Aza-dC at day 1–2, 3–5, or 6–8, and cells were harvested at day 8 of the differentiation. We found that inhibiting DNA methylation by 5-Aza-dC at the early differentiation stage from day 1–5 inhibited adipogenesis, evident by decreased mRNA expression of adipogenic markers such as PPARγ2, CEBPα, and aP2, (Fig. 1A). However, the inhibitory effect of 5-Aza-dC disappeared when cells were treated with 5-Aza-dC at the late stage of differentiation (day 6–8) (Fig. 1A). Similar inhibition of PPARγ at protein levels was also observed in 3T3-L1 cells treated with 5-Aza-dC at the early stage of differentiation at day 1–2 (Fig. 1B). Oil Red O staining revealed less lipid droplet accumulations in 3T3-L1 cells treated with 5-Aza-dC at the early differentiation stage of day 1–2 (Fig. 1C). These data suggest that inhibition of DNA methylation by 5-Aza-dC at the early stage of differentiation suppresses adipogenesis.

### The Wnt10a expression is up-regulated by 5-Aza-dC in 3T3-L1 cells

**Figure 2:**
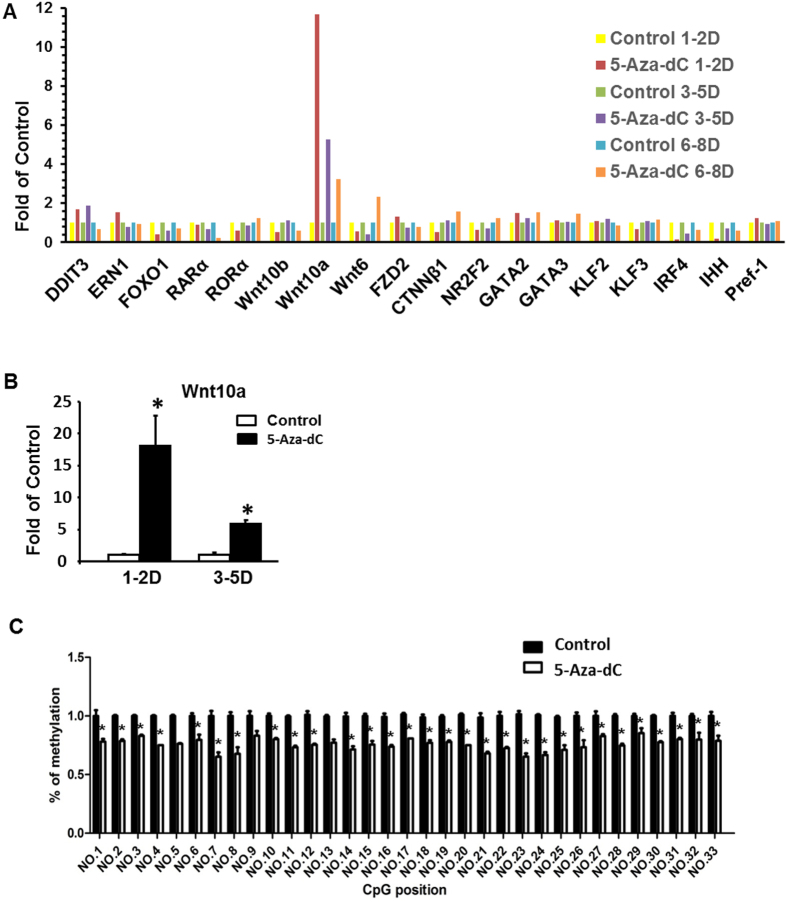
The Wnt10a gene is demethylated and up-regulated by 5-Aza-dC in 3T3-L1 cells. (**A**) Real-time PCR screening on repressor gene expression of 3T3-L1 cells treated with 5-Aza-dC during adipogenesis. (**B**) Wnt10a mRNA expression is dramatically induced by early treatment of 5-Aza-dC. (**C**) Pyrosequencing analysis showed that 5-Aza-dC treatment significantly reduces DNA methylation levels on all the 33 CpG sites measured in 3T3-L1 cells at day 1–2 of differentiation. 3T3-L1 preadipocyte were induced to differentiate with a differentiation medium as described in Materials and Methods, and were concurrently treated with either PBS or 5-Aza-dC at the time points as indicated in the figure. For (**A**,**B**), gene expression was measured by real-time RT-PCR and normalized to cyclophilin. For (**A**), n = 3–6 samples were pooled for one real-time PCR measurement. For (**B**), all data are expressed as mean ± SEM, n = 4. For (**C**), CpG methylation was measured by pyrosequencing as described in the Materials and Methods, n = 4. All data are expressed as mean ± SEM, *p < 0.05 vs. control. Con: control; AZA: 5-aza-dC.

To further explore the mechanism underlying the suppression of adipogenesis by the early inhibition of DNA methylation with 5-Aza-dC, we surveyed the expression of a number of key transcriptional factors that control the adipogenic program. Since demethylation of gene promoters by 5-Aza-dC activates gene expression and the early treatment of 5-Aza-dC led to inhibition of adipogenesis in our study, we reckoned that the anti-adipogenic transcriptional factors might be 5-Aza-dC’s potential targets, activation of which may be responsible for the suppression of adipogenesis by the treatment of 5-Aza-dC during early differentiation. Among the adipogenic repressors we screened using a group of pooled samples (n = 3–6 samples pooled for one real-time PCR measurement), Wnt10a mRNA expression was dramatically induced by early treatment of 5-Aza-dC, with a peak level reached at day 1–2 (Fig. 2A). We confirmed this result by measuring Wnt10a expression in individual samples (Fig. 2B). This evidence suggests that Wnt10a may play a key role in inhibiting adipocyte differentiation by 5-Aza-dC treatment.

The Wnt10a 5′ untranslated region including part of the first exon is enriched with CpG sites, raising the possibility that Wnt10a may be regulated by DNA methylation. Among approximate 46 CpG sites in the 5′ region, we chose to measure the methylation status of the 33 CpG sites located on the first exon region before the start codon (Supplemental Fig. 1). Using pyrosequencing analysis, we found that DNA methylation levels were significantly reduced on all 33 CpG sites measured in 3T3-L1 cells treated with 5-Aza-dC at day 0–2 of differentiation (Fig. 2C). These data suggest that demethylation of Wnt10a gene by 5-Aza-dC may cause Wnt10a overexpression, leading to suppression of adipogenesis during the early stage of differentiation.

**Figure 3:**
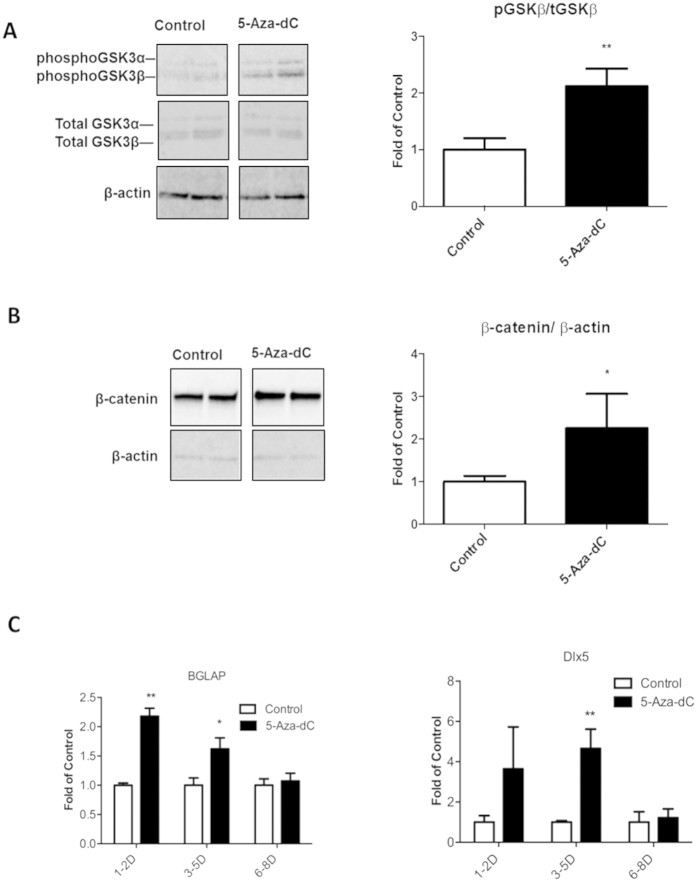
Inhibition of DNA methylation by 5-Aza-dC at the early stage of differentiation activates Wnt/β-catenin signaling and enhances gene expression in osteoblastogenesis in 3T3-L1 cells. (**A**) Treatment of 5-Aza-dC increases phosphorylation of GSK3β at the early stage of differentiation. (**B**) Treatment of 5-Aza-dC increases β-catenin content at the early stage of differentiation. (**C**) Treatment of 5-Aza-dC enhances the expression of osteoblastogenic markers BGLAP and Dlx5. 3T3-L1 preadipocyte were induced to differentiate with a differentiation medium as described in Materials and Methods, and were concurrently treated with either PBS or 5-Aza-dC at day 1 of differentiation (in **A**,**B**) and at the time points indicated in (**C**). mRNA and protein levels were measured by real-time RT-PCR and immunoblotting, respectively. Representative blots are shown in (**A**,**B**). All data are expressed as mean ± SEM (n = 3–6); *p < 0.05; **p < 0.01 vs. control.

We further examined phospho-GSK3 and β-catenin levels, and found that both phospho-GSK3β and β-catenin protein content were up-regulated in 3T3-L1 cells treated with 5-Aza-dC at the early differentiation stage (Fig. 3A,B). Since Wnt/β-catenin signaling is important in the determination of adipogenic vs. osteobalstogenic fate^5^,^10^, we tested whether activation of Wnt/ β-catenin signaling would increase osteoblastogenesis in the 3T3-L1 cells treated with 5-Aza-dC. Indeed, the early treatment of 5-Aza-dC enhanced the expression of osteoblastogenic markers such as Bone Gamma-Carboxyglutamate (Gla) Protein/ Osteocalcin (BGLAP) and Distal-Less Homeobox 5 (Dlx5) (Fig. 3C). These data suggest that inhibiting DNA methylation by 5-Aza-dC increases Wnt10a expression by demethylating the 5′ untranslated region of the gene, which in turn activates Wnt/ β-catenin signaling, leading to reciprocal down-regulation of adipogenesis and up-regulation of osteoblastogenesis.

### Inhibition of DNA methylation by 5-Aza-dC enhances osteobalstogenesis in ST2 stem cells

**Figure 4:**
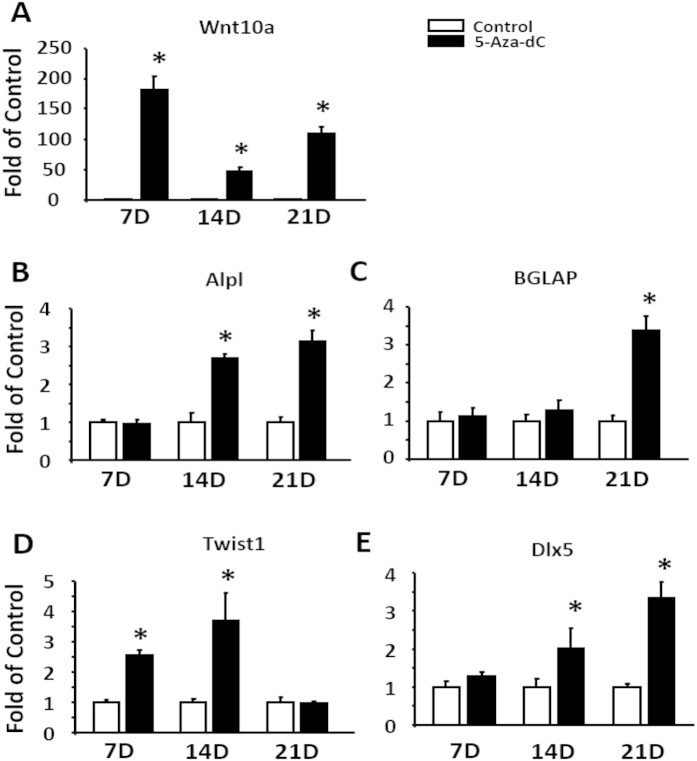
Inhibition of DNA methylation by 5-Aza-dC enhances the expression of osteoblastogenic markers in ST2 stem cells. Treatment of 5-Aza-dC increases the mRNA expression of Wnt10a (**A**) and osteoblastogenic markers Alpl (**B**), BGLAP (**C**), Twist1 (**D**), and Dlx5 (**E**). ST2 cells were induced to differentiate into osteoblasts in the presence of either PBS or 5-Aza-dC at the time points indicated in the figures. mRNA was measured by real-time RT-PCR. All data are expressed as mean ± SEM (n = 3–6); *p < 0.05 vs. control.

**Figure 5:**
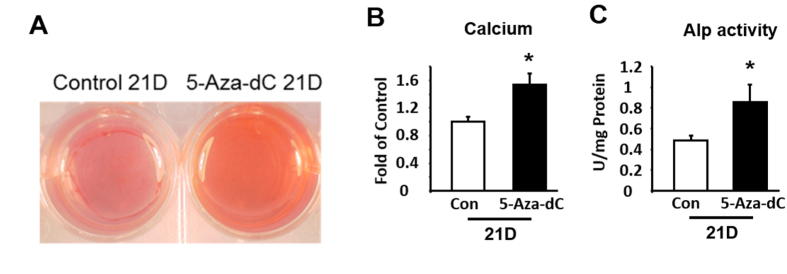
Inhibition of DNA methylation by 5-Aza-dC promotes osteoblastogenesis in ST2 stem cells. Treatment of 5-Aza-dC increases calcium content, measured by Alizarin Red staining (**A**) and quantitation (**B**), and alkaline phosphatase activity (**C**). ST2 cells were induced to differentiate into osteoblasts in the presence of either PBS or 5-Aza-dC at the time points indicated in the figures. Alizarin Red staining and alkaline phosphatase activity measurement were conducted as described in the Materials and Methods. Representative images are shown in (**A**). All data are expressed as mean ± SEM (n = 3–6); *p < 0.05 vs. control.

To further determine the role of DNA methylation in regulation of osteoblastogenesis, we used ST2 cells, a mesenchymal stem cell model that has a pluripotent capacity to develop into either adipocytes or osteoblasts depending upon differentiation conditions. ST2 cells were induced to differentiate into osteoblasts in the presence of either PBS or 5-Aza-dC. We found that treatment of 5-Aza-dC markedly induced mRNA expression of Wnt10a during osteoblastogenesis (Fig. 4A). This was associated with up-regulation of the expression of osteoblastogenic markers such as alkaline phosphatase (Alpl), BGLAP, Basic Helix-Loop-Helix Transcription Factor 1 (Twist1) (an early osteoblastogenic marker^18^), and Dlx5 (Fig. 4B–E). We further confirmed osteoblastogenic phenotype in differentiated ST2 cells by measuring calcium content and Alkaline Phosphatase activity. Treating ST2 cells with 5-Aza-dC for 21 days during osteoblastogenesis significantly increased calcium content, as measured by Alizarin red staining and quantitation (Fig. 5A,B). Similar results were observed on alkaline phosphatase activity in ST2 cells treated with 5-Aza-dC (Fig. 5C).

### Activation of the Wnt signaling mediates the anti-adipogenic effect of 5-Aa-dC in 3T3-L1 cells

**Figure 6:**
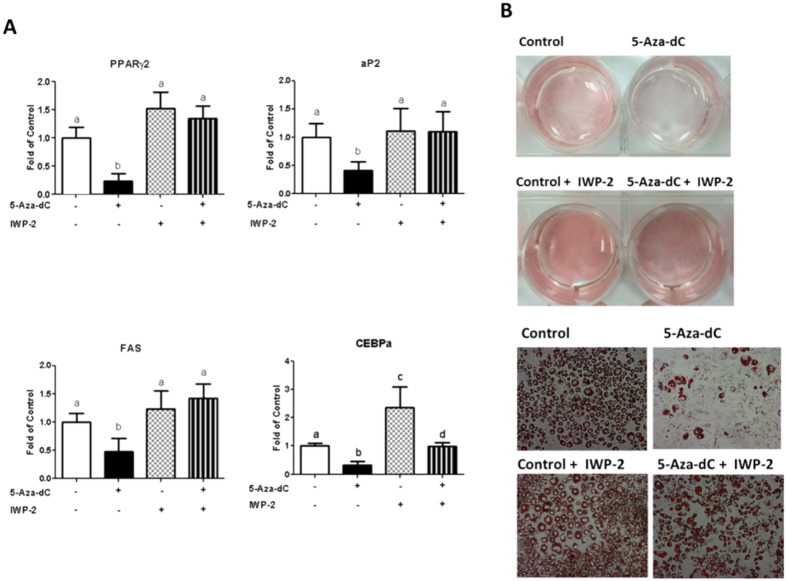
Activation of the Wnt signaling mediates the anti-adipogenic effect of 5-Aza-dC in 3T3-L1 cells. (**A**) Treatment of IWP-2, a Wnt inhibitor, largely prevents the inhibitory effect of 5-Aza-dC on the expression of adipogenic markers PPARγ, aP2, FAS and C/EBPα. (**B**) IWP-2 largely prevents the inhibitory effect of 5-Aza-dC on lipid accumulation measured by Oil Red O staining. 3T3-L1 cells were induced to differentiate and were concurrently treated with either PBS or 5-Aza-dC in the presence or absence of IWP-2 at day 1–2 of the differentiation period. Gene expression was measured by real-time RT-PCR and normalized to cyclophilin. Oil Red O staining was conducted as described in the Materials and Methods. Representative images are shown in (**B**). All data are expressed as mean ± SEM (n = 3–6); groups labeled with different letters are statistically different from each other.

To determine whether activation of Wnt signaling mediates the anti-adipogenic effect of 5-Aza-dC, we blocked Wnt signaling with a pharmacological approach in 3T3-L1 cells. We chose to use IWP-2, an inhibitor of Wnt protein production^19^. 3T3-L1 cells were induced to differentiate with an adipogenic cocktail and were concurrently treated with either PBS or 5-Aa-dC in the presence or absence of IWP-2 at day 1–2 of the differentiation period. Interestingly, treatment of IWP-2 completely reversed the inhibitory effect of 5-Aza-dC on the expression of PPARγ, aP2 and fatty acid synthase (FAS) mRNA levels and partially blocked the inhibitory effect of 5-Aza-dC on the expression of C/EBPα mRNA level (Fig. 6A). Moreover, Oil Red O staining revealed less lipid droplet accumulation in 3T3-L1 cells treated with 5-Aza-dC at day 1–2 of the early differentiation stage, which was completely reversed by IWP-2 treatment (Fig. 6B). These data suggest that inhibition of the Wnt signaling by IWP-2 prevents the anti-adipogenic effect of 5-Aa-dC in 3T3-L1 cells.

### Activation of the Wnt signaling mediates the pro-osteoblastogenic effect of 5-Aa-dC in ST2 cells

**Figure 7:**
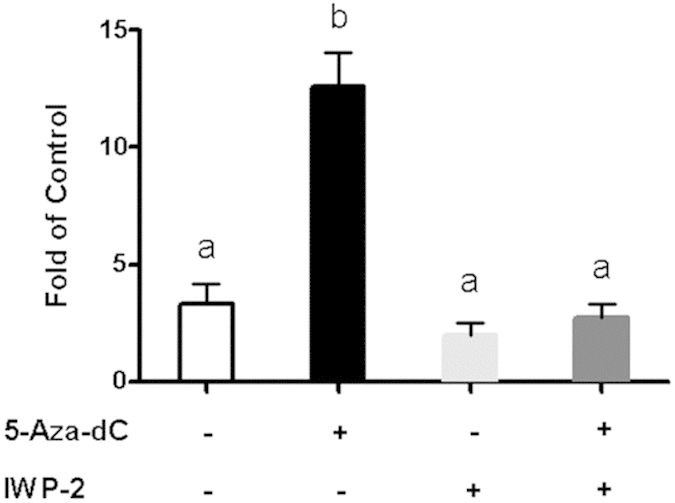
Activation of the Wnt signaling mediates the osteoblastogenic effect of 5-Aza-dC in ST2 cells. IWP-2 prevents the stimulatory effect of 5-Aza-dC on the expression of the osteoblastogenic marker BGLAP. ST2 stem cells were induced to differentiate with an osteoblastogenic regimen and were concurrently treated with either PBS or 5-Aza-dC in the presence or absence of IWP-2 in the differentiation period. ST2 cells were harvested at day 21 after fully differentiated into osteoblasts. Gene expression was measured by real-time RT-PCR and normalized to cyclophilin. All data are expressed as mean ± SEM (n = 3–6); groups labeled with different letters are statistically different from each other.

We further determined whether activation of Wnt signaling mediates the pro-osteoblastogenic effect of 5-Aza-dC in ST2 cells. ST2 stem cells were induced to differentiate with an osteoblastogenic regimen and were concurrently treated with either PBS or 5-Aza-dC in the presence or absence of IWP-2 in the differentiation period. We found that treatment of IWP-2 completely reversed the inhibitory effect of 5-Aza-dC on the expression of the osteoblastogenic marker BGLAP (Fig. 7). These data suggest that inhibition of the Wnt signaling by IWP-2 prevents the osteoblastogenic effect of 5-Aa-dC in ST2 cells.

### Inhibition of DNA methylation by 5-Aza-dC promotes osteoblastogenic conversion in the adipocyte lineage 3T3-L1 cells

**Figure 8:**
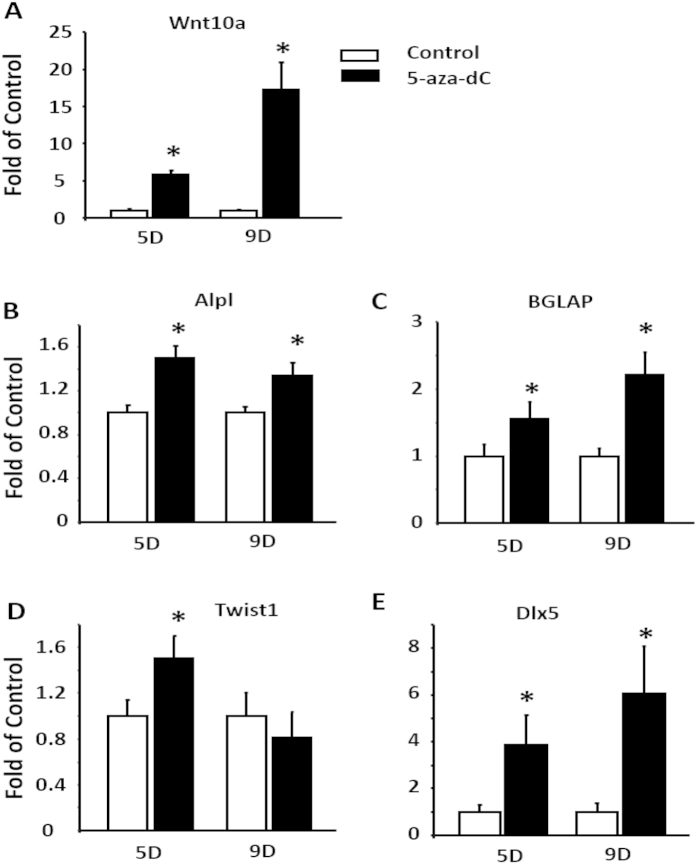
Inhibition of DNA methylation by 5-Aza-dC promotes osteoblastogenic conversion in adipocyte lineage 3T3-L1 cells. Treatment of 5-Aza-dC stimulates the expression of Wnt10a (**A**) and osteoblastogenic markers Alp (**B**), BGLAP (**C**), Twist1 (**D**), and Dlx5 (**E**) in 3T3-L1 cells subjected to osteoblastogenic differentiation. 3T3-L1 preadipocytes were induced to differentiate with an osteoblastogenic regimen and were concurrently treated with either PBS or 5-Aa-dC at the time pints of the differentiation period as indicated in the figures. Gene expression was measured by real-time RT-PCR and normalized to cyclophilin. All data are expressed as mean ± SEM (n = 3–6); *p < 0.05 vs. control.

3T3-L1 fibroblasts are committed preadipocytes belonging to adipocyte lineage cells^20^, which are presumably difficult to be differentiated into osteoblasts via lineage conversion. To determine the ability of 5-Aza-dC to promote osteoblastogenesis, we adopted the adipocyte lineage cells 3T3-L1 to study osteoblastogenic process. 3T3-L1 cells were induced to differentiate with an osteoblastogenic regimen and were concurrently treated with either PBS or 5-Aa-dC during the differentiation period. As expected, treatment of 5-Aza-dC stimulated the expression of Wnt10a in 3T3-L1 cells subjected to osteoblastogenic differentiation (Fig. 8A). This was associated with up-regulation of osteoblastogenic markers Alpl, BGLAP, Twist1 and Dlx5 (Fig. 8B–E). These data suggest that 5-Aza-dC displays a strong ability of lineage conversion to promote osteoblastogenesis.

## Discussion

Both Adipocytes and osteoblasts originate from the mesenchymal stem cells^1^,^2^. The signaling pathways regulating lineage determination of the mesenchymal stem cells have received intense investigation. Recent data suggest that Wnt/β-catenin mutual-exclusively determines adipogenic or osteoblastogenic lineage^5^,^10^. In the present study, we demonstrate that DNA methylation plays an important role in determining adipogenic versus osteoblastogenic lineage through regulating Wnt10a gene. Our data indicate that inhibiting DNA methylation by 5-Aza-dC at the early stage of differentiation suppresses adipogenesis, while promoting osteoblastogenesis presumably through stimulating Wnt10a expression by demethylating its promoter.

We demonstrate in this study that DNA methylation orchestrates the adipogenic or osteoblastogenic program through Wnt10a. We actually observed a transient but profound induction of DNA methyltransferase 1 (DNMT1), a key enzyme for maintaining DNA methylation, within 48 hours of the early adipogenic stage (data not shown and will be reported elsewhere). The unique expression pattern of DNMT1 during early adipogenesis is extremely interesting and may dictate the terminal fate of adipogenesis distinguished from osteoblastogenesis. Methylation of gene promoter by DNMTs typically results in gene silencing. We reasoned that the surge of DNMT1 expression at the beginning of adipogenesis may silence the early adipogenic repressor Wnt10a, which may be critical in determining adipogenic fate^10^. Indeed, we found that inhibiting DNA methylation by 5-Aza-dC demethylates the Wnt10a promoter and subsequently induces its expression, leading to the suppression of adipogenic program and the simultaneous promotion of osteoblastogenic program. The anti-adipogenic and pro-osteoblastogenic effects of 5-Aa-dC can be largely reversed by a pan-Wnt inhibitor IWP-2. It is noteworthy that several Wnt family proteins such as Wnt10a, Wnt10b, and Wnt6 have been shown to mutual-exclusively regulate the terminal fate of osteoblastogenesis versus adipogenesis^10^. Interestingly, a number of Wnt ligands such as Wnt4, Wnt5a, Wnt 5b, Wnt 7b, Wnt 9b and Wnt11 are subjected to DNA methylation^21^. We found that only Wnt10a was significantly increased in 5-Aza-dC treated 3T3-L1 and ST2 cells, but not other Wnt members. Therefore, inhibition of DNA methylation by 5-Aza-dC treatment may mainly target Wnt10a during 3T3-L1 adipogenesis and ST2 osteoblastogenesis. Nonetheless, additional studies involving genetic approaches are required to identify and confirm 1) which DNMTs are responsible for the anti-adipogenic and osteoblastogenic effects of 5-Aa-dC; 2) which Wnt ligand(s) mediates these effects of 5-Aa-dC. On the other hand, it would be interesting to know whether DNMTs also act on other gene promoters to regulate transcription during adipogenesis and osteoblastogenesis.

Most studies investigating the mechanisms underlying the regulation of adipogenesis and osteoblastogenesis focus on transcriptional pathways; little is known about the epigenetic mechanisms in this process. Epigenetic regulation, including DNA methylation, is a molecular link between environmental factors (e.g. diets) and complex diseases, including obesity and diabetes. This is a new emerging research area. However, evidence converges to suggest that epigenetic events figure prominently in adipogenesis^14^,^15^. Using genome-wide epigenetic mapping, Mikkelsen *et al*. have found regulated changes of histone methylation/acetylation marks during adipogenesis^22^. These dynamic changes of histone marks may actively regulate adipogenesis through interaction with key transcriptional activators or repressors that control adipogenic program. Indeed, the transcriptional active mark H3K4me has been shown to promote adipogenesis through interacting with the promoter of PPARγ^14^. In addition, Wnt proteins have been identified as epigenetic targets during adipogenesis. For example, histone methyltransferase G9a deletion decreases the methylation at the repressive histone mark histone 3 lysine 9 (H3K9) at Wnt10a promoter, leading to Wnt10a overexpression and subsequent inhibition of adipogenesis^23^. Further, evidence suggests that histone 3 lysine 27(H3K27) methylation inhibits adipogenesis through de-repressing the Wnt family proteins^14^. Changes in DNA methylation have been shown to modulate histone methylation, which may act cooperatively to influence chromatin structure and thereby regulate gene expression^24^. This raises another interesting question as to whether DNA methylation also regulates adipogenesis and osteoblastogenesis through modulating histone methylation. Additional studies are warranted to investigate the potential crosstalk between DNA methylation and histone modification in the reciprocal regulation of adipogenesis and osteoblastogenesis.

It is noteworthy that the DNA methylation inhibitor 5-Aza-dC has been used to convert embryonic stem cells C3H10T1/2 into mesodermal stem cell lineages that can further differentiate into myocytes, chondrocytes and adipocytes^25^,^26^. Further studies from Dr. Daniel Lane’s group demonstrated that a subpopulation of 5-Aza-dC-induced C3H10T1/2 cells have a strong propensity to differentiate into adipocyte upon treatment with an adipogenic differentiation regimen^27^. The discrepancy between their observations and our findings are not clear. One possibility might be the different cell models at different developmental stages that are used for differentiation in these published studies. C3H10T1/2 cells are a kind of embryonic stem cells that presumably have pluripotent potentials to develop into diverse cell types^1^, while either 3T3-L1 preadipocytes or bone marrow-derived ST2 cells^28^ are more committed cell types in the realm of the mesodermal lineage. Therefore, the ability of 5-Aza-dC to suppress adipogenesis and promote osteoblastogenesis may only occur in more committed cell types. Nonetheless, a more recent report that investigated the role of 5-Aza-dC in osteogenesis in adipose-derived stem cells is consistent with our findings^29^. Yan *et al*. recently demonstrated that 5-Aza-dC promotes osteogenesis in human adipose-derived mesenchymal cells by DNA demethylation^29^.

Adipogenesis contributes to the development of obesity^30^,^31^, which was thought be protective against osteoporosis due to the beneficial effect of enhanced mechanical loading from the excessive weights^32^,^33^,^34^. The positive effect of obesity on bone mineral density^35^ was questioned by other large population studies showing no positive correlation between bone mass and BMI^34^,^36^,^37^. Instead, recent epidemiological studies demonstrated that increased fat mass particularly visceral fat is associated with osteoporosis^32^,^33^. The underlying mechanisms linking increased adiposity to bone loss involve the endocrine and immune functions of adipose tissue, which secretes numerous adipokines and cytokines^32^,^33^. These fat-derived factors during the development of obesity are detrimental to the bone formation^32^. Another potential link between obesity and bone loss may reside on the bone marrow mesenchymal stem cells (MSCs), the common precursors capable of becoming both adipocytes and osteoblasts^32^,^38^. It is noteworthy that the fate of MSCs is regulated by signaling pathways such as Wnt/β-catenin that mutual-exclusively determines adipogenic or osteoblastogenic lineage^5^,^10^. Our study demonstrates an epigenetic mechanism controlling the commitment of MSCs into either adipocytes or osteoblasts via modulation of DNA methylation at the Wnt10a promoter. Future studies are warranted to determine whether obesity-related factors such as pro-inflammatory cytokines can increase the promoter methylation of Wnt10a, resulting in diminished capacity of MSCs to become osteoblasts and leading to osteoporosis.

In summary, we demonstrate that inhibiting DNA methylation by 5-Aza-dC at the early stage of differentiation significantly inhibits adipogenesis in 3T3-L1 cells whereas promoting osteoblastogenesis. 5-Aza-dC demethylates Wnt10a gene, leading to enhanced Wnt10a expression, which likely mediates the anti-adipogenic and pro-osteoblastogenic effect of 5-Aza-dC in 3T3-L1 and ST2 cells. We conclude that DNA methylation plays a significant role in lineage determination of adipogenesis versus osteoblastogenesis. Our study defines DNA methylation as a novel mechanism underlying adipocyte and bone cell development. The findings could also help guide the development of epigenetic regulation as new therapeutic targets in the prevention and treatment of obesity and/or osteoporosis.

## Methods

### Adipogenesis

3T3-L1 preadipocytes (ATCC, Manassas, VA) were grown in a DMEM medium containing 10% new born calf serum (NBCS) and 5% penicillin. Two days after confluence, 3T3-L1 preadipocytes were induced to differentiate with DMEM containing 10% fetal bovine serum (FBS), 0.5 mM 3-isobutyl-1-methylxanthine, 1 μM dexamethasone, and 400 nM insulin. Cells were incubated in this medium for two days and then cultured in growth medium containing 400 nM insulin for another 2 days. Subsequently, cells were maintained in growth medium (without insulin) throughout the adipocyte stage and cultures were refed every two days. Cells were treated with either PBS or 5-Aza-dC (0.5 μM) at different time points indicated in the figures and were harvested at day 8 of the differentiation.

### Osteoblastogenesis

To induce osteoblastogenesis, 3T3-L1 or ST2 cells were grown and incubated in a DMEM low glucose medium containing 10% FBS and 5% Penicillin solution. Two days after confluence (post-confluence), 3T3-L1 preadipocytes or ST2 stem cells (designated as day 0) were cultured with a DMEM containing 10% FBS, 10 mM β-glycerophosphate and 20 μg/ mL ascorbic acid-2-phosphate. The osteoblastogenic medium was replaced every 2 days afterwards. Cells were treated with either PBS or 5-Aza-dC throughout the differentiation process and were harvested at different points indicated in the figures. In some experiments, ST2 cells were treated with either PBS or 5-Aza-dC in the presence or absence of 10 mM IWP-2 and were harvested at different time points indicated in the figures.

### Oil red O staining

Accumulation of lipids in adipocytes was assessed by Oil Red O staining. Oil Red O was purchased from Sigma-Aldrich (St. Louis, MO) and was prepared as 0.5% solution in isopropanol. Cells were fixed with 10% formaldehyde for at least 1 hour and washed twice with water. Oil red O working solution was prepared in the ratio of 40% water and 60% Oil red O, left at least 20 minutes, filtered and then added to fixed cells. Cells were incubated at room temperature for 10 minutes and then washed 4 times with water.

### Alizarin Red staining

The degree of mineralization in osteoblasts was determined using Alizarin Red staining^39^. Alizarin Red S (catalog # A5533) was purchased from Sigma-Aldrich (St. Louis, MO) and was prepared as 2% solution in water. The solution was filtered and added to fixed cells. Cells were incubated with the Alizarin Red solution at room temperature for 30 minutes. The Cells were then de-stained for quantification by adding 1 mL 5% perchloric acid and the absorbance was read in a 96 wells plate reader at 490 nm.

### Measurement of alkaline phosphatase activity

The activity of alkaline phosphatase (Alp) in osteoblasts was measured using an Acid Phosphatase Assay Kit (catalog # CS0740) purchased from Sigma-Aldrich (St. Louis, MO). Cells were lysed by using a lysis buffer (1% v/v Igepal^®^ CA-630; 10 mM Tris-HCl; 1 mM MgCl_2_, pH 7.5) for overnight at −80 °C. Lysates then were centrifuged at 12,000 rpm for 10 minutes and 50 μl of supernatant was then used to measure Alp activity using the Acid Phosphatase Assay Kit according to manufacturer’s instruction.

### Total RNA extraction and quantitative RT-PCR

Total RNA of the 3T3-L1 cells and ST2 cells was extracted using the Tri Reagent kit (Molecular Research Center, Cincinnati, OH), according to the manufacturer’s protocol. The expression of genes of interest was assessed by quantitative RT-PCR (ABI Universal PCR Master Mix, Applied Biosystems, Foster City, CA) using a Stratagene Mx3005p thermocycler (Stratagene, La Jolla, CA), as we previously described^40^,^41^. The primer and probe pairs used in the assays were purchased from Applied Biosystems (Foster City, CA).

### Immunoblotting

Immunoblotting was conducted as we previously described^40^. Cells were harvested and homogenized in a modified radioimmunoprecipitation assay (RIPA) buffer containing 50 mM Tris (pH 7.4), 1 mM EDTA (pH 8.0), 150 mM NaCl, 1% NP-40, 0.5% sodium deoxycholate, 1 mM each of NaF, NaVO_3_ and PMSF, 1% of protease inhibitor cocktail (Sigma-Aldrich) and 1% each of serine and tyrosine phosphatase inhibitor cocktail (Sigma-Aldrich). Lysates were centrifuged at 15,000 rcf for 30 min at 4 °C and then supernatants were transferred to fresh tubes and stored at −80 °C. Approximately 20–50 μg of protein was run on 4–15% precast polyacrylamide gel and transferred to PVDF membrane. Membranes were blocked in 5% non-fat and then immunoblotted with the indicated primary antibodies (1:200~1:1000 dilution, each in 2% non-fat milk) overnight at 4 °C, and fluorescence-conjugated secondary antibodies Alexa Fluor 680 (1:5000 or 1:10000 dilution in 5% non-fat milk) at room temperature for 2 hrs. The blots were developed with a Li-COR Odyssey Imager system (Li-COR Biosciences, Lincoln, NE). Alexa Fluor 680-conjugated secondary antibodies were purchased from Invitrogen (Carlsbad, CA). Mouse β-catenin antibody (catalog # sc-7199), mouse β-actin antibody (catalog # sc-1616), PPARγ antibody (catalog # sc-7196 or sc-7273) was purchased from Santa Cruz Biotechnology (Dallas, TX). Rabbit Phospho-GSK-3α/β antibody (catalog # 9331) was purchased from Cell Signaling (Danvers, MA). Mouse Anti-GSK, clone 4G-1E antibody (catalog # 05-412) was purchased from Millipore (Billerica, MA). Polyacrylamide gel (catalog # 5671084) and Immun-Blot^®^ PVDF membrane (catalog # 1620177) were purchased from Bio-Rad (Hercules, CA).

### Bisulfite conversion and pyrosequencing

Genomic DNA was prepared by phenol/chloroform extraction. Bisulfite conversion was performed using EpiTech Bisulfite Kit (Qiagen, Valencia, CA). The converted DNA was used as template to amplify DNA sequence covering the putative CpG sites at the Wnt10A or SREBP1C promoter regions. Approximately 1μg of bisulfite converted DNA was amplified by PCR and pyrosequencing was performed by EpiGenDx (Hopkinton, MA). The PCR and pyrosequencing primers that were used to measure the DNA methylation on the Wnt10a 5′ region covering CpG sites are described in Supplemental Table 1.

### Statistics

All data are expressed as mean ± SEM. Data were evaluated for statistical significance by one way ANOVA, and statistical significance for comparison of means of different groups was calculated by the least-significant-difference test using the SPSS software package version 11.5. p < 0.05 was considered significant.

## Additional Information

**How to cite this article**: Chen, Y.-S. *et al*. Inhibiting DNA methylation switches adipogenesis to osteoblastogenesis by activating Wnt10a. *Sci. Rep*. **6**, 25283; doi: 10.1038/srep25283 (2016).

## Supplementary Material

Supplementary Information
