# Supplementary material for: Inhibiting DNA methylation switches adipogenesis to osteoblastogenesis by activating Wnt10a
Source: Sci Rep. 2016 May 3;6:25283. doi: 10.1038/srep25283 (PMC4853709; doi:10.1038/srep25283)

Inhibiting DNA methylation switches adipogenesis to osteoblastogenesis by activating Wnt10a

Yii-Shyuan Chen, Rui Wu, Xiaosong Yang, Shuping Kou, Ormond A. MacDougald, Liqing Yu, Hang Shi and Bingzhong Xue

Supplemental Table 1. Amplification and Sequencing primers for Wnt10a

Primers	Sequences
Amplification primer 1: Forward	5’- GGTGGGGTTGGGTTAGATAGT-3’
Amplification primer 1: Reverse	5’- ACTACCACACACAACTCCTACTCA-3’
Amplification primer 2: Forward	5’- AGGGGTTTTTGGGAAATTTTTTG-3’
Amplification primer 2: Reverse	5’- ACTACCACACACAACTCCTACTCA-3’
Amplification primer 3: Forward	5’- TGTTGAGGGAGTTGTGATTTGAGTAG-3’
Amplification primer 3: Reverse	5’- CCTAAAAAATCCTAACTCTCCAAAAAAAC-3’
Sequencing primer 1	5’- GGTTGGGTTAGATAGTAT-3’
Sequencing primer 2	5’- GTTGGGGTGGGGGGT-3’
Sequencing primer 3	5’- ATTTTTGTGTTAGGAGG-3’
Sequencing primer 4	5’-ACTCCTACTCAAATCAC-3’
Sequencing primer 5	5’- TGTGATTTGAGTAGGAG-3’
Sequencing primer 6	5’-AGTTTTTTATTTTTGGTTTGTT-3’

Supplemental Figure Legends

Supplemental Figure 1. Schematic illustration of the Wnt10A promoter and 5'-end region. The transcription start site is indicated as +1, while the translation start site is shown as ATG. TATA box, C/EBP, CAAT box and exon 1 are indicated. The 33 CpG cites measured in this study are indicated as upward vertical lines in exon 1. F1/R1, F2/R2, and F3/R3 represent the forward and reverse primers of amplification RCR for pyrosequencing analysis.

Supplemental Figure 1

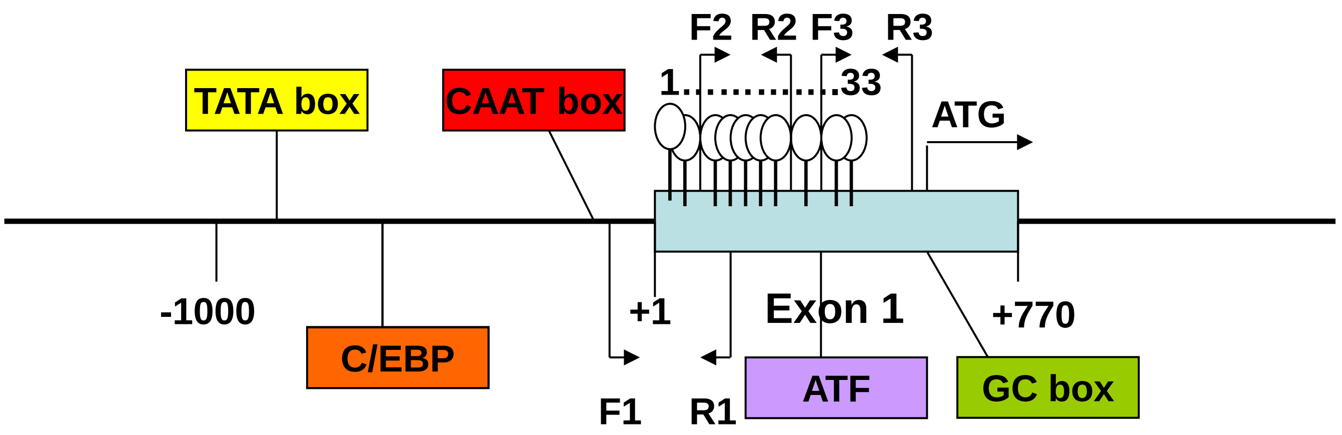

Supplement: Supplementary Information [file srep25283-s1.pdf]
